# Case report: intracranial lesions in a patient with anxiety and depression: tumor recurrence or radiation encephalopathy?

**DOI:** 10.3389/fonc.2024.1422765

**Published:** 2024-08-15

**Authors:** Haiping You, Lin He, Zhibo Ouyang, Yao Yang, Shu Xie, Jiwei Zhou, Yun Zhang, Jian Shi

**Affiliations:** ^1^ Department of Psychosomatic Medicine, Mianyang Central Hospital, School of Medicine, University of Electronic Science and Technology of China, Mianyang, China; ^2^ Department of Clinical Medicine, Chengdu Medical College, Chengdu, China

**Keywords:** radiation encephalopathy, anxiety disorders, depressive disorders, nasopharyngeal cancer, radiotherapy

## Abstract

**Purpose:**

Radiation encephalopathy (REP) is one of the most common complications of radiotherapy for malignant tumors of the head and neck. Symptoms usually appear months to years following radiotherapy, with headache, insomnia, and memory loss as the main clinical features. We report a patient who was admitted to the hospital with anxiety and depressive disorder and was eventually diagnosed with REP.

**Patients and methods:**

A 48-year-old patient who had undergone over 2 years of radiotherapy for nasopharyngeal carcinoma was admitted to the Department of Psychosomatic Medicine of our hospital because of recurrent fear, low mood, and waking up from dreams. Magnetic resonance imaging (MRI) revealed a mass in the left temporal lobe with a large peripheral edema. After multidisciplinary consultation, the possibility of tumor recurrence could not be excluded.

**Results:**

Resection of the lesioned brain tissue to obtain pathological tissue showed glial cell proliferation and small focal areas of degeneration and necrosis, which indicated that the lesions were inflammatory. Postoperative MRI showed no abnormal signal, and the patient’s condition improved.

**Conclusion:**

Nasopharyngeal carcinoma patients with a history of radiotherapy and symptoms of increased intracranial pressure and neurological damage should be examined for REP. Furthermore, patients may experience anxiety and depressive disorders as a result of temporal lobe damage caused by REP.

## Introduction

In China, nasopharyngeal cancer is one of the most common malignant tumors of the head and neck. Owing to the anatomical location and radiosensitivity of nasopharyngeal cancers, radiotherapy is considered the first-line treatment (1). Although radiotherapy is effective, it damages the body in varying degrees, with some patients developing Radiation encephalopathy (REP). REP, also known as radiation-induced brain injury, is one of the most common complications of radiotherapy for head and neck malignant tumors that typically develops months to years following radiotherapy. It is predominantly characterized by headache, insomnia, and emotional disorders, and REP usually occurs in the temporal lobe (2). The average crude incidence of temporal lobe necrosis in particular in the REP is approximately 14% (3). Although brain metastasis of nasopharyngeal carcinoma is the rarest mode of metastasis, both metastasis and recurrence are the most common causes of death in these patients (4). Therefore, brain metastasis cannot be excluded when patients present with symptoms of elevated intracranial pressure and neurological injury. We report a 48-year-old patient who was admitted to the hospital with anxiety and depressive disorder. After multidisciplinary consultation and treatment, the brain lesion was removed via craniectomy, and the patient was diagnosed with REP based on pathology results. He was discharged from the hospital after his condition improved following the surgery.

## Case presentation

A 48-year-old patient was admitted to our hospital on March 23, 2023, because of fear, depression, and over 2 months of insomnia. The patient had been hospitalized over 2 months prior because of Coronavirus Disease 2019. During hospitalization, the patient’s father died, and the patient began experiencing recurrent episodes of fear, difficulty falling asleep, nightmares, surging pain throughout the body, occipital pain, toothache, and pain paroxysms. These symptoms worsened in the week before admission. Symptoms worsened after activities, and the patient gradually became depressed and lost interest in activities he enjoyed previously. He also developed memory loss, slowed response, and poor concentration. When the symptoms were severe, the patient felt tired all day and was not interested in talking, going out, or socializing with others. The patient cried occasionally, felt excessively nervous, worried about trivial matters, and was irrationally fearful, which stopped him from going out. The patient scored 25 points on the Hamilton Anxiety Scale (HAMA), 20 points on the Hamilton Depression Scale (HAMD), and 24 points on the Mini-mental State Examination (MMSE), which indicated significant anxiety and depression symptoms and mild cognitive impairment.

The patient had a nasopharyngeal carcinoma over 2 years ago, which had been treated with radiotherapy and antitumor therapy, and had been taking dexzopiclone for over 2 years. 60Gy/33f was administered during nasopharyngeal cancer treatment. The patient received gemcitabine plus cisplatin chemotherapy, which is the standard of care for nasopharyngeal carcinoma, 80 mg/m2 cisplatin (every 3 weeks), and 1 g/m2 gemcitabine (two intravenous doses on D1 and D8) every 3 weeks. The patient’s symptoms of the primary nasopharyngeal cancer lesion resolved after radiotherapy. Because of the preliminary anxiety and depressive disorder diagnosis, the patient underwent cranial MRI and magnetic resonance angiography (MRA) to detect brain lesions. The patient was prescribed 0.5 to 1 mg hora somni lorazepam and 10 mg quaque die escitalopram, and changes in condition were monitored.

**Figure 1:**
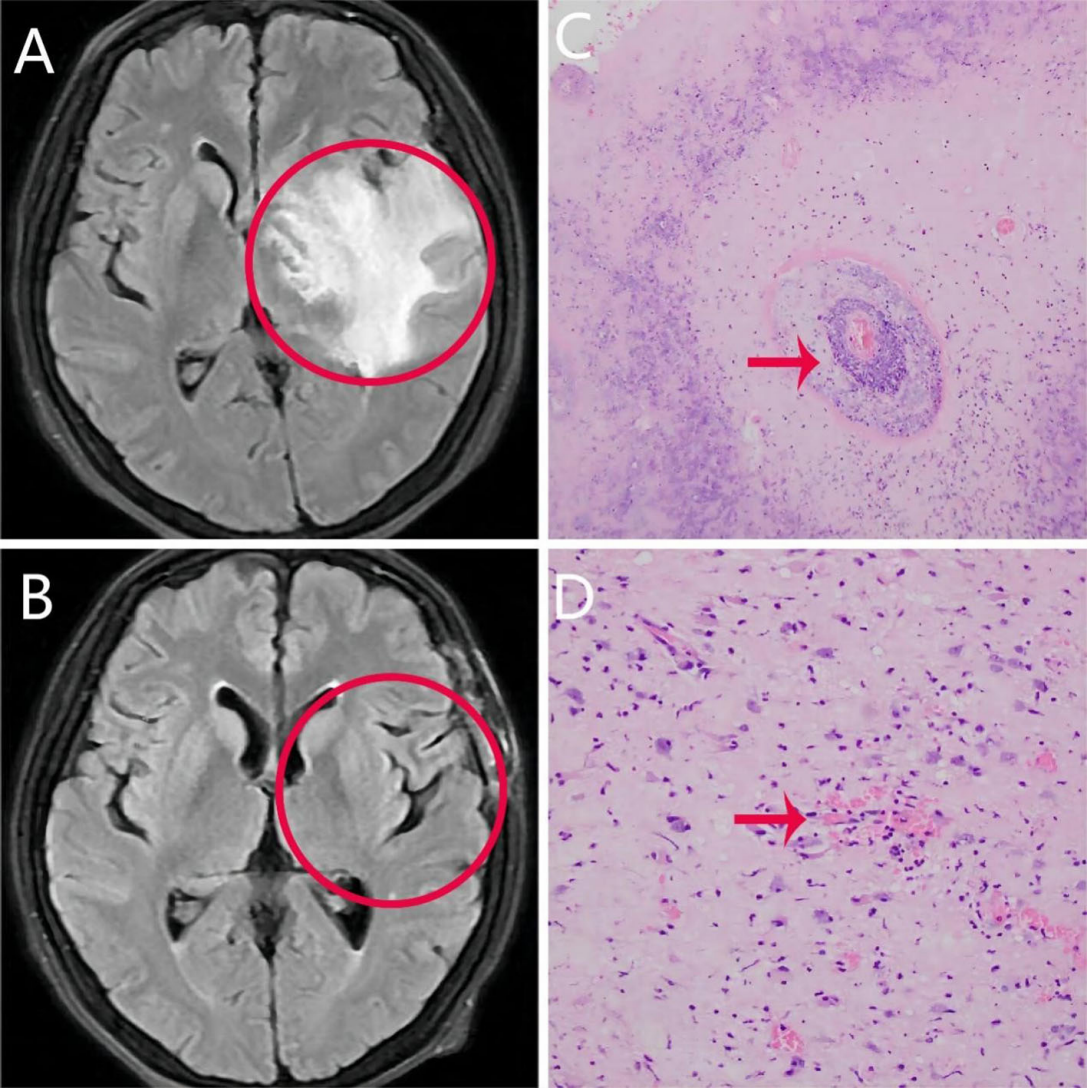
Clinical pictures of patients. **(A)** Cranial T2 fluid-attenuated inversion recovery sequence magnetic resonance imaging scan showing an irregular mass of abnormal signal in the anterior portion of the left temporal lobe. The borders between the mass and surrounding tissue are not clear. **(B)** Abnormal signal disappeared after resection of lesioned tissue. **(C, D)** Brain histopathology showing extensive degeneration and necrosis of glial cells, hyperplasia of peripheral glial cells, and infiltration of peripheral lymphoid tissue.

Cranial MRI scans (T2 fluid-attenuated inversion recovery sequence; **Figure 1A**) showed a left temporal lobe mass with large surrounding edema and midline shift. No obvious abnormalities were detected in the cerebral white matter on high-signal magnetic resonance venogram (MRV) and MRA scans. Because of intracranial hypertension, the neurologist recommended administration of mannitol to lower brain pressure. Oncology consultation was requested, and cranial direct enhancement and magnetic resonance spectroscopy (MRS) were recommended. Cranial direct enhancement and spectral imaging showed an irregular mass-like abnormal signal in the anterior portion of the left temporal lobe, and direct enhancement showed clear inhomogeneous wreath-like enhancement with unclear borders. The adjacent meninges of the skull base was thickened, markedly enhanced, and surrounded by a large edema band involving the left frontal lobe and basal ganglia. The adjacent left lateral ventricle and part of the sulcus were compressed, and the midline structure deviated to the right by approximately 1.1 cm. The right temporal lobe also showed significantly enhanced nodules, with a size of approximately 0.7 × 0.5 cm. MRS of the left temporal lobe lesion showed reduced N-acetylaspartate (NAA) and creatine (Cr) peaks. Additionally, the choline (Cho) peak was slightly decreased, the NAA/Cr value was significantly decreased, and the Cho/Cr value was increased. This suggested a high probability that the left temporal lobe had radiological damage.

Pathologic findings of the patient’s primary lesion showed: poorly differentiate squamous cell carcinoma (non-keratinizing). Based on the summarized WHO histologic types can be defined as type 2 (non-keratinizing squamous) (5). The patient reported significant improvement in sleep and a slight reduction in fear. However, because of the radiotherapy for nasopharyngeal carcinoma, the patient continued to experience headaches and double vision. These headaches occurred when he changed position, and they lasted for approximately 30 seconds. Because abnormalities were detected in the cranial MRI, neurosurgery consultation was requested, and surgery was recommended for the treatment of cerebral edema and intracranial hypertension. After obtaining consent from the patient and his family, the patient was referred to the neurosurgery department to undergo the operation. The postoperative primary lesion on the repeat MRI (**Figure 1B**) disappeared, and brain histopathology (**Figures 1C, D**) showed extensive degeneration and necrosis of glial cells, hyperplasia of peripheral glial cells, and infiltration of peripheral lymphoid tissues, which led to a final diagnosis of REP. After the craniotomy and subsequent care, the patient’s condition improved and he was thus discharged. During outpatient follow-up in November 2023, the patient showed improvements in both anxiety and depressive symptoms (HAMA score: 13 points; HAMD score: 10 points; MMSE score: 26 points). Antidepressant medication had been discontinued, and apart from sleep disturbances, the patient had no other symptoms. During a follow-up in January 2024 over the phone, the patient reported significant improvement and had no anxiety, depression, sleep problems, or generalized surge pain.

## Discussion

Previous studies have reported that patients with nasopharyngeal cancer have a higher incidence and degree of anxiety and depression during radiotherapy (6). Anxiety disorder is a common mental illness in which patients experience unexplained preoccupation, nervousness, significant autonomic dysfunction symptoms, muscle tension, and motor restlessness. Symptoms include alertness, irritability, sensitivity to stimuli, fidgetiness, inattentiveness, and excessive worrying. However, the pathogenic mechanism underlying anxiety disorders remains unclear. The development of anxiety disorders is associated with changes in brain structure, which may be detected via brain imaging. Patients with generalized anxiety disorder show changes in amygdala and temporal lobe volumes. Furthermore, four brain regions—the left inferior temporal gyrus and medial temporal lobe, supplementary motor area, thalamus, and anterior cingulate cortex—are strongly associated with posttraumatic cognitive impairment and psychiatric symptoms (7), which is consistent with REP. Structural changes in the temporal lobe, frontal lobe, and other regions during the development of anxiety disorders have a causal effect on generalized anxiety disorder (8). Moreover, anxious individuals have higher amplitude of low-frequency fluctuation values in various subcortical structures, including the striatum, thalamus, medial temporal lobe, midbrain, pons, and cerebellum (9). In patients with temporal lobe epilepsy, depression is associated with decreased temporal lobe metabolism (10). In our patient, MRI showed a peripheral mass in the left temporal lobe with large peripheral edema, which resulted in volume changes in the temporal lobe and, in turn, symptoms of anxiety and depression. It is worth noting, however, that the influence of psychological factors cannot be excluded.

The unique pathological characteristics of nasopharyngeal cancer make these tumors more sensitive to radiotherapy. Thus, the first-line treatment method for nasopharyngeal cancer is radiotherapy. With the improvement of radiotherapy technology, the 5-year survival rate of nasopharyngeal cancer patients has significantly increased. However, REP has now become the most serious complication of nasopharyngeal cancer following radiotherapy, and the lack of accurate clinical and imaging features for REP has presented difficulties in early diagnosis and treatment. Previous studies have shown that among nasopharyngeal cancer patients, the incidence of bone metastasis is the highest, whereas the incidence of brain metastasis is the lowest (11). In addition, recurrence and metastasis are the most common causes of death (4). In one study, local recurrence occurred in 14% of patients, whereas distant metastasis occurred in 21% of patients with local recurrence (12). We can make a rough differential diagnosis based on symptoms and signs. Patients with brain metastases tend to present with headaches, dizziness, motor deficits, sensory deficits, and cranial neurologic symptoms (13), while temporal lobe injuries tend to be characterized by dizziness, mood disorders, and cognitive dysfunction (14). Our patient presented with headaches, depression, sleep disturbances, and anxiety symptoms, alongside a left temporal lobe mass with large peripheral edema. Although the edema around the lesion was a feature of acute brain radionecrosis due to REP (15), it was not possible to accurately differentiate radiological encephalopathy from primary tumor metastasis. Therefore, in nasopharyngeal carcinoma patients with a history of radiotherapy, tumor metastasis should be prioritized when intracranial hypertension and neurological symptoms are present. While we removed the tumor, the pathology showed that it was REP. This requires us to pay more attention to the effects of radiation therapy.

Symptoms of REP are caused by brain damage or disease due to radiation exposure (16). Common symptoms are headaches and cognitive deficits, such as memory loss, poor concentration, and slowed thinking. Neuropsychological evaluation is crucial to identify neurological symptoms, mood swings, depression, anxiety, irritability, and behavioral abnormalities, some of which may lead to epileptic seizures (15, 17). REP is typically observed in the temporal lobe. Temporal lobe dysfunction occurs in neuropsychiatric and related disorders because the temporal lobe interacts directly with the limbic system and plays an important role in cognition, emotion regulation, the autonomic nervous system, and information transmission (18). Our patient had damage to the left temporal lobe due to prolonged radiotherapy, which may have contributed to the patient’s mood changes, anxiety, and depression.

The diagnosis of REP in patients with nasopharyngeal carcinoma is primarily based on MRI. Structural and functional MRI is more sensitive than conventional MRI for identifying radiation-induced brain damage (19). Contrast-enhancing lesions are found in all injured temporal lobes and are the most common MRI abnormalities detected in patients with REP (20). A previous study summarized three models of radiological temporal lobe injury: (1) white matter lesions (with uniformly high signal intensity on T2-weighted imaging (WI) and low signal intensity on T1WI), (2) contrast-enhancing lesions (high signal intensity on T2WI and postcontrast enhancement on T1WI), and (3) cysts (rounded lesions with a thin wall or those that are difficult to visualize, with very high signal intensity on T2WI). A diagnosis of radiation-induced temporal lobe injury is confirmed when at least one of the three models is detected on MRI and these features are not caused by other factors, such as tumor metastasis (2). Our patient’s MRI of the left temporal lobe was consistent with the abovementioned imaging features and thus was confirmed as radiation-induced temporal lobe injury. The final diagnosis of REP was made after obtaining these findings. It has previously been shown that radiotherapy causes a dramatic decrease in the cortical volume of certain areas of the temporal, occipital, and frontal lobes; the left temporal lobe in particular shows a dose-dependent decrease in cortical volume (21). Radiotherapy can induce structural changes in certain brain regions, which can be detected as abnormalities in brain imaging. MRS is more sensitive in identifying tumor recurrence and radionecrosis because it measures the levels of various metabolites in brain tissue (22–24). It has been found that elevated Cho peaks in tissue after treatment indicate residual or recurrent tumors with high specificity. However, the absence of elevation does not exclude residual or recurrent tumors; in fact, the Cho/Cr values obtained from MRS data are more relevant to tumors (25). In patients with stable disease, the metabolic ratios of MRS data are significantly negatively correlated with NAA/Cr and NAA/Cr(h) values and are not associated with tumors (25, 26). In our patient, data showed high cerebral white matter signal and contrast enhancement in the lesioned area, which satisfied one of the above models. Furthermore, the MRS data revealed a slightly lower Cho peak, a markedly lower NAA/Cr value, and a higher Cho/Cr value, which suggested that both tumor metastasis and REP were possible. However, because the decrease in NAA/Cr value was more pronounced, REP was deemed more likely. Taken together, our findings suggest that white matter lesions, contrast-enhancing lesions, and cysts are three models that are not only applicable to temporal lobe injuries but also useful for diagnosing REP with the aid of MRS.

Pathological examination is the gold standard for the diagnosis of REP. However, given the potential complications of obtaining tissue, the high cost, invasiveness, and large sampling error (15, 23), surgical resection is only considered in patients who remain symptomatic despite conservative treatment or those with an uncertain diagnosis. The main advantages of surgical resection are the relief of any lesion occupancy and histological confirmation (27). Biopsied or excised tissues are fixed in 10% formalin, processed and examined in hematoxylin-eosin-stained paraffin sections using immunostaining. The percentage of the area of surviving persistent/recurrent tumor, radionecrosis, and normal brain tissue in all sections of each resection specimen is estimated via retrospective examination of all relevant sections (24). Histologically, brain radiation injury is characterized by the presence of geographic eosinophilic necrosis with atypical glial cell proliferation, peripheral lymphocytic infiltration, and associated inflammation (28). Tumor progression is characterized by the predominant presence of mitotically active tumor cells. Typically, in metastatic tumors surgically resected specimens, histological features of cerebral radionecrosis and tumor progression may coexist, and diagnosis is based on the predominant lesion feature (15). In our patient, the biopsy showed extensive degeneration and necrosis of glial cells, peripheral glial cell hyperplasia, and peripheral lymphoid tissue infiltration, which are features that are most typical of radiation-induced brain injury. Thus, the final clinical diagnosis was REP.

In addition to surgical treatment, REP can also be treated adjunctively with medications, mostly steroid, bevacizumab, etc. Steroid treatment has been established as the mainstay of treatment for REP patients with mild or no symptoms. A Study have shown that steroid treatment does not affect the survival of patients without complications (29). Bevacizumab, in addition to being used as a targeted therapy for certain cancers, can also be used to treat REP including temporal lobe injury. A controlled trial of bevacizumab and cortisol in REP patients found that bevacizumab was more significant in symptomatic relief and improvement in edema and enhancement on MRI (30). However, it may cause adverse events such as hypertension, so the treatment of bevacizumab needs to be further studied.

## Conclusion

Radiotherapy is a crucial treatment method for nasopharyngeal cancer. The most common complication following radiotherapy for nasopharyngeal cancer is radiation-induced brain injury, and the temporal and frontal lobes are the most vulnerable to damage. Anxiety disorders and depression disorders are common symptoms of radiation-induced temporal lobe injury. These symptoms can be relieved by reducing cerebral edema via surgical treatment.

## Data Availability

The raw data supporting the conclusions of this article will be made available by the authors, without undue reservation.
